# Highly Selective Methodology for Entrapment and Subsequent Removal of Cobalt (II) Ions under Optimized Conditions by Micellar-Enhanced Ultrafiltration

**DOI:** 10.3390/molecules27238332

**Published:** 2022-11-29

**Authors:** Amnah Yusaf, Muhammad Usman, Matloob Ahmad, Muhammad Siddiq, Asim Mansha, Sami A. Al-Hussain, Magdi E. A. Zaki, Hafiza Fatima Rehman

**Affiliations:** 1 Department of Chemistry, Government College University, Faisalabad 38000, Pakistan; 2 Department of Chemistry, University College London, London WC1E 6BT, UK; 3 Department of Chemistry, Government College Women University, Faisalabad 38000, Pakistan; 4 Department of Chemistry, Quaid-i-Azam University, Islamabad 45320, Pakistan; 5 Department of Chemistry, College of Science, Imam Mohammad Ibn Saud Islamic University (IMSIU), Riyadh 13623, Saudi Arabia; 6 Department of Zoology, Government College University, Faisalabad 38000, Pakistan

**Keywords:** partition coefficient, binding constant, micelles, metal ions, adsorption, MEUF, rejection percentage, permeate flux, concentration of surfactant, electrolyte, transmembrane pressure, RPM, pH

## Abstract

Micellar-enhanced ultrafiltration (MEUF), being a separation technique, was used to remove cobalt metal ion (Co^2+^) from their aqueous solutions in an application to reduce the toxicity level from industrial effluents using a micellar solution of anionic and cationic surfactants. The metal ions were first adsorbed by using anionic surfactants, i.e., sodium dodecyl sulfate (SDS) and sodium oleate (SO). The calculations for partition (K_x_) and binding constants (K_b_) and their respective free energy of partition and binding (ΔG_p_ and ΔG_b_ kJmol^−1^) helped significantly to find out the extent of binding or interaction of Co^2+^ with the surfactant and ΔG_p_ and ΔG_b_ were found to be −29.50 and −19.38 kJmol^−1^ for SDS and −23.95 and −12.67 kJmol^−1^ in the case of SO. MEUF work was also performed to find out the optimal conditions to remove metal pollutants from the aqueous system. For the said purpose, various factors and concentrations effect were studied, such as the concentration of the surfactant, concentration of the electrolyte (NaCl), transmembrane pressure, RPM, and pH. The efficiency of this process was checked by calculating various parameters, such as rejection percentage (R%) and permeate flux (J). A maximum rejection of 99.95% with SDS and 99.99% with SO was attained.

## 1. Introduction

The word “heavy metals” implies any metallic element that has relatively higher density and is very poisonous, even if its concentration is very low. Environmental pollution due to said metals is a main concern in modern societies. Lead (Pb), arsenic (As), chromium (Cr), cobalt (Co), etc., are usually termed heavy metals. In numerous applications, these heavy metal ions may enter into the water bodies by industrial and chemical plants, i.e., textile, electroplating, metal mines, tanneries, and paints, for which they are required for their effective working [1,2,3]. Due to the rapid industrialization in developed countries, the sewage of various industries is using significant amounts of toxic metal ions [4,5,6]. Additionally, textile production is one of the most water-consuming units of industries due to its high water consumption [7]. Due to the use of heavy metals in these industries, polluted water becomes a serious threat to the environment and injurious to human health [8]. These metals produce an imbalance in living organisms by developing several mechanisms, particularly in humans, resulting in a wide range of disorders. These ions are not only carcinogenic, but also cause nervous disorders, skin diseases, blood disorders, cardiovascular diseases, kidney damage and mass accumulation in tissues [5,9].

The removal of such metal ions requires different chemical and physical methods, such as ion exchange, precipitation, flocculation, and adsorption. Such conventional methods are very costly and consume a lot of labor energy [10]. MEUF is safer, and requires low cost and energy compared to the traditional techniques, as it requires low working pressure and also membranes can be reused. As far as concerns regarding the surfactant cost, the surfactants, soaps, are already present in the textile effluent. MEUF is capable of removing multivalent ions due to the electrostatic interaction present between the micelles and the metal cations, and hence, a high yield of rejection percentage can be attained [9].

The use of surfactants or their micellar media for removing metal ions is really a need of the hour and can successively be removed by anionic surfactants as reported in the literature [9].

Surfactants (surface active agents) are an exclusive class of organic compounds, composed of two particular groups: a hydrophobic tail (water-insoluble) and a hydrophilic head (water-soluble). Thus, they are amphiphilic in nature [11]. Generally, the chain structure of surfactants is mainly attributed to a hydrophobic tail consisting of a long fluorocarbon chain, a siloxane chain, a hydrocarbon chain, or a short polymer chain [12]. On the other hand, the head group consists of sulfates, sulfonates, polyoxymethylene chains, carboxylates, alcohols, or quaternary ammonium salts. The inclusion of these moieties reveals the unique and fascinating behavior of surfactants, which has led to their daily usage, from households to the industrial field as a wetting agent, foaming agent, detergents, flotation, solubilizers, emulsifiers, drug delivery, and oil recovery [13]. At a critical micellar concentration, the amphiphilic nature of surfactants in an aqueous solution allows the formation of colloidal particles (micelles) by self-aggregation and minimizes Gibbs free energy by arranging on the water–air interface [14]. The polarity of the micellar environment decreases from the surface to the inner core in aqueous media [15]. After interfacial adsorption, when the surfactant concentration is gradually increased, micellization is the only way for the stabilization of the system. The spontaneity of micellization is due to noncovalent forces, which are hydrogen bonding, van der Waal forces, or hydrophobic interaction. Micelles are extensively used in vitro studies (drug–surfactant interaction) due to exterior hydrophilic and interior hydrophobic interactions, which grant them resemblance to the bio-membrane [16]. The solubilization and detergency process is directly analogous to micellization. Solubilization is the spontaneous formation of the stable isotropic solution by increasing the solubility of the water-insoluble or sparingly soluble compound by using micellar media. Particularly, it is the distribution of aqueous insoluble compounds between the bulk and micellar phase via dynamically reversible interactivity following Nernst’s distribution law [17,18,19].

Adsorption is also a useful method due to its efficiency, and the diverse membrane-based techniques exploited for wastewater treatment include reverse osmosis (RO), adsorptive ultrafiltration (UF), nanofiltration (NF), flocculation, coagulation, and microfiltration (MF) [20,21,22]. Conventional ultrafiltration (UF) is magnified in micellar-enhanced ultrafiltration (MEUF) which employs surfactants above CMC. Hence, among all the techniques, micellar enhanced ultrafiltration has proven to be a wonderful methodology [23,24,25,26].

MEUF has been found to be a well-investigated surfactant-based promising technique to remove metal ions, organic pollutants or other contaminants, as it possesses the phenomena of high flux ultrafiltration as well as the reverse osmosis (RO) process [27,28,29]. The removal of metal ions by micellar-enhanced ultrafiltration can be done in three phases. (i) The micelles are formed by surfactants, and the negatively charged hydrophilic part rests at the interface, whereas the hydrophobic region oriented into the core, (ii) metal ions or other impurities of a cationic nature, binds at the charged surface. (iii) The solution is passed through a ultrafiltration cell with a membrane containing small pores and thus the ability to block the passage of micelle-containing pollutants [30]. Therefore, this method is beneficial because of the high rejection percentage of pollutants, low operational cost and energy consumption as well as high rate of flow [31].

Cobalt chloride (II), being a toxic metal, is often used in industries in the manufacturing of glues, adhesive materials, and sealants and also for the electroplating of objects or surface treatment. This metal results in eye irritation and other diseases, and even a minute amount can even cause death. This metal is used in various industries, and hence, their industrial wastewater containing metals and other pollutants comes to our domestic sewage, water bodies lines, land, crops, plants, etc., resulting in severe damage to the whole system. The present research aimed to study the partitioning of metal Co^2+^ in diverse micellar media by UV-Visible spectroscopy [32]. The consequence of a micellar medium of anionic surfactants on metal was investigated on small scale; hence, to modify the mechanism, more innovation in this work is still needed, although some work has been reported for the solubilization of pollutants by a simple and mixed system as well as for the removal of metals and other pollutants but scant data are available to sort out the optimal conditions and factors for a low-cost and highly effective frame [33]. In this research, the heavy metal pollutant (Co^2+^) is first adsorbed at the surface and then ultrafiltered by MEUF. We expect that this study will be supplementary and supportive of the selection of premier micellar media as well as the suitable optimal conditions, such as metal, surfactant and electrolyte concentration, including such factors as transmembrane pressure, RPM, and pH for spontaneous metal encapsulation.

## 2. Results and Discussions

### 2.1. UV-Visible Spectroscopy

#### 2.1.1. Simple and Differential Absorption Spectra

**Figure 1:**
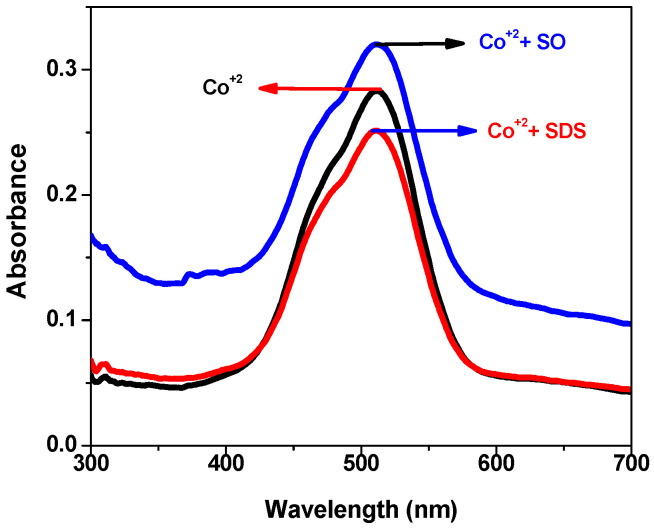
Simple absorption spectra of CoCl_2_ in the presence and absence of surfactant.

**Figure 2:**
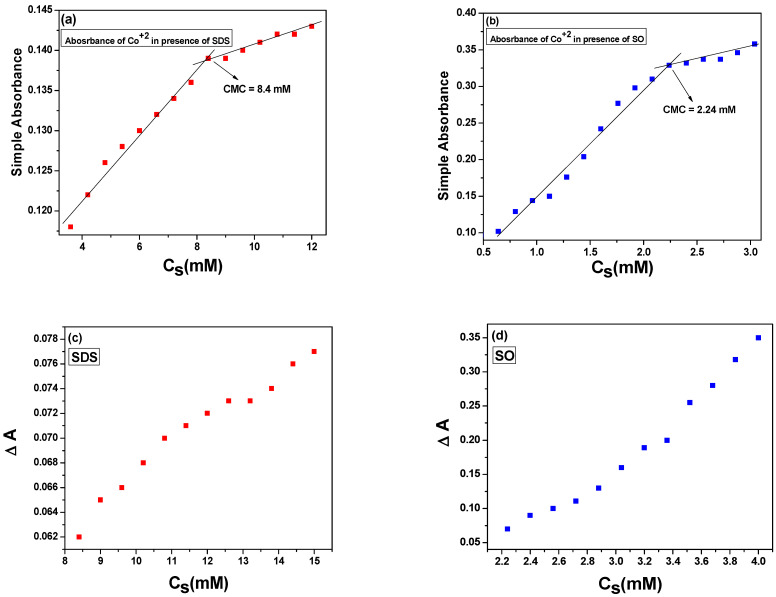
(**a**) Plot for simple absorbance of CoCl_2_ as function of SDS concentration. (**b**) Plot for simple absorbance of CoCl_2_ as function of SO concentration. (**c**) Plot for differential absorbance of CoCl_2_ as function of SDS concentration. (**d**) Plot for differential absorbance of CoCl_2_ as function of SO concentration.

In the presence and absence of both anionic surfactants, the simple absorption spectra of metal (Co^2+^) are shown in Figure 1. The addition of a surfactant to the solution of Co^2+^ results in the re-stabilization and redistribution of energy levels and simple absorption spectra of CoCl_2_ as a function of the SDS and SO concentrations, shown in Figure 2a,b. The critical micellar concentration (CMC) was found to be 8.4 mM and 2.24 mM for SDS and SO, respectively, having a close agreement to the literature reported values as 8.23 mM and 2.14 mM, respectively, for SDS and SO [34,35]. Because of the incessant adsorption of cations at the micellar heads, a rise in the differential absorbance is shown in Figure 2c,d [36,37].

#### 2.1.2. Interaction of Co^2+^ in SDS and SO Systems

UV/Visible spectra of CoCl_2_ solution (1 × 10^2^ M) in the presence and absence of surfactants showing maximum absorption at 512 nm as shown in Figure 1. The opposite charges of metal and surfactant leads to the formation of metal–surfactant complexes [38,39] as the aggregated surfactant monomers known as micelles and metal ions tend to be absorbed by these micelles due to the presence of electrostatic or van der Waals forces [40]. The charged groups on the surface, therefore, attract counter ions in the solution through electrostatic interaction [2,41]. The absorption of Co^2+^ as a function of the concentration of the surfactant is displayed in Figure 2. Surfactants produce hypsochromic shifts in spectra, owing to electrostatic or van der Waals forces between the cationic nature of metal and the anionic surfactants to form aggregates of metal and surfactant. After adsorption, no difference was absorbed in λ_max_ of Co^2+^ at the surfaces, confirming that the chromophores stayed in the polar surroundings. A regular rise in absorbance occurs by increasing the surfactant concentration because of the interaction of the surfactant and metal.

These aggregates do not carry on for a long time and are broken due to the adsorption of Co^2+^ molecules at the micellar surface at the CMC point. At CMC, due to the Co^2+^ adsorption in newly made micelles, the absorbance does not achieve a constant value but increases slowly [42].

**Scheme 1:**
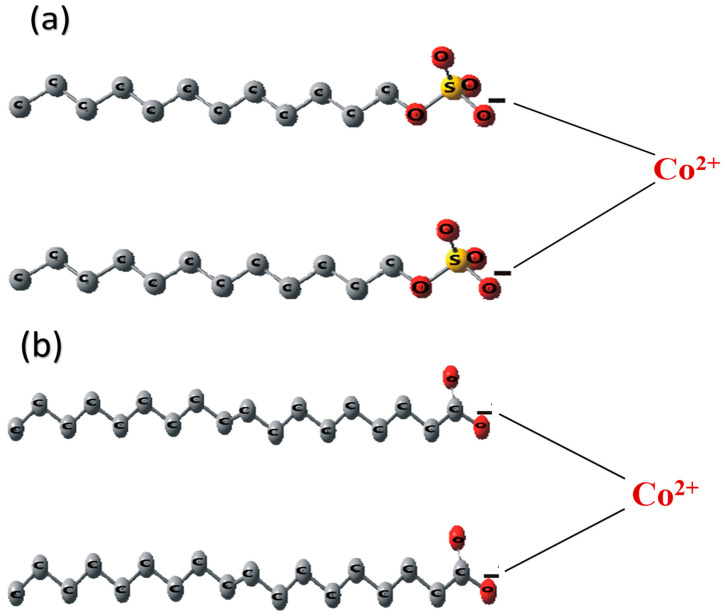
Insight into dye–surfactant interaction (**a**) Co^2+^-SDS (**b**) Co^2+^-SO.

When the surfactant concentration is away from the CMC point, the molecules of surfactant try to accumulate themselves into micelle form and bind with metal cations in order to form bulky surfactant and metal structures. Initially, the ion pairs of metal and surfactant stay close to the surfactant–water boundary and later on move to become adsorbed at the micellar surface because of the addition of a surfactant to the liquid and has the ability to adjust the surface properties [43]. A pictorial insight into the dye–surfactant interaction is shown in Scheme 1.

**Figure 3:**
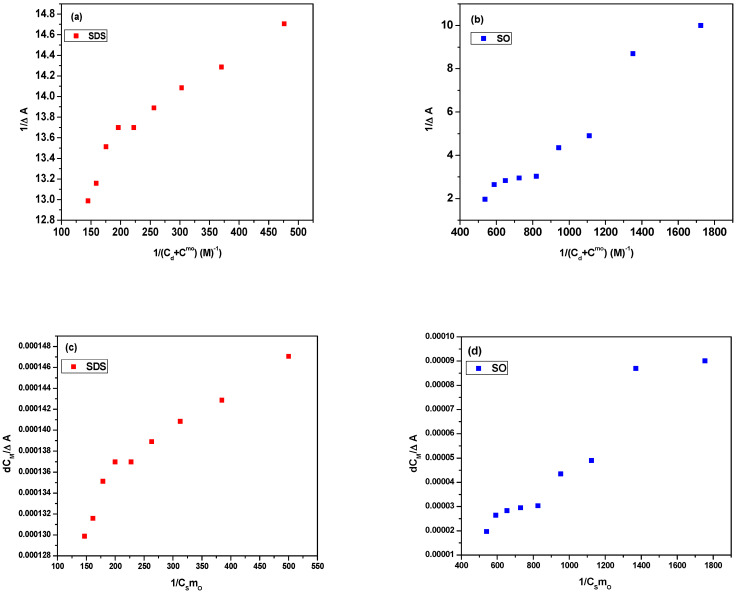
(**a**) Kawamura plot for calculation of partition constant, K_x_, for the Co^2+^/SDS system (**b**) Kawamura plot for calculation of partition constant, K_x_, for the Co^2+^/SO system (**c**) Benesi–Hildebrand plot for the calculation of binding constant, K_b_, for the Co^2+^/SDS system. (**d**) Benesi–Hildebrand plot for calculation of binding constant, K_b_, for the Co^2+^/SO system.

The data of some important parameters were calculated and are given in Table 1. The plots for the calculation of binding and partition constant of Co^2+^ in SDS and SO are shown in Figure 3a–d. Below the CMC point, the differential absorbance of metal ions is zero, as the micelles are not present. The partition and binding coefficients for the Co^2+^/SDS system and the Co^2+^/SO system were calculated by the Gibbs free energy of partition, ΔG_p_ (−29.50 kJmol^−1^ for Co^2+^SDS system and −23.95 kJmol^−1^ for Co^2+^/SO system), whereas for Gibbs energy of binding, ΔG_b_ (−19.38 kJmol^−1^ for Co^2+^/SDS system and −12.67 kJmol^−1^ for Co^2+^/SO system). The negative value ΔG_p_ is an indication of the spontaneous process [12,44].

#### 2.1.3. Interaction of Co^2+^ in SDS/SO System

This study showed a strong binding of Co^2+^ with single SDS micelles because of the sulfate group present in SDS having high charge density due to rare charge delocalization, so, consequently, it can accommodate a larger number of metal cations. Additionally, due to the COO^−^ group present in SO, the binding of SO with metal ions was noticed.

### 2.2. MEUF for the Removal of Single Metal Ion (Co^2+^)

**Scheme 2:**
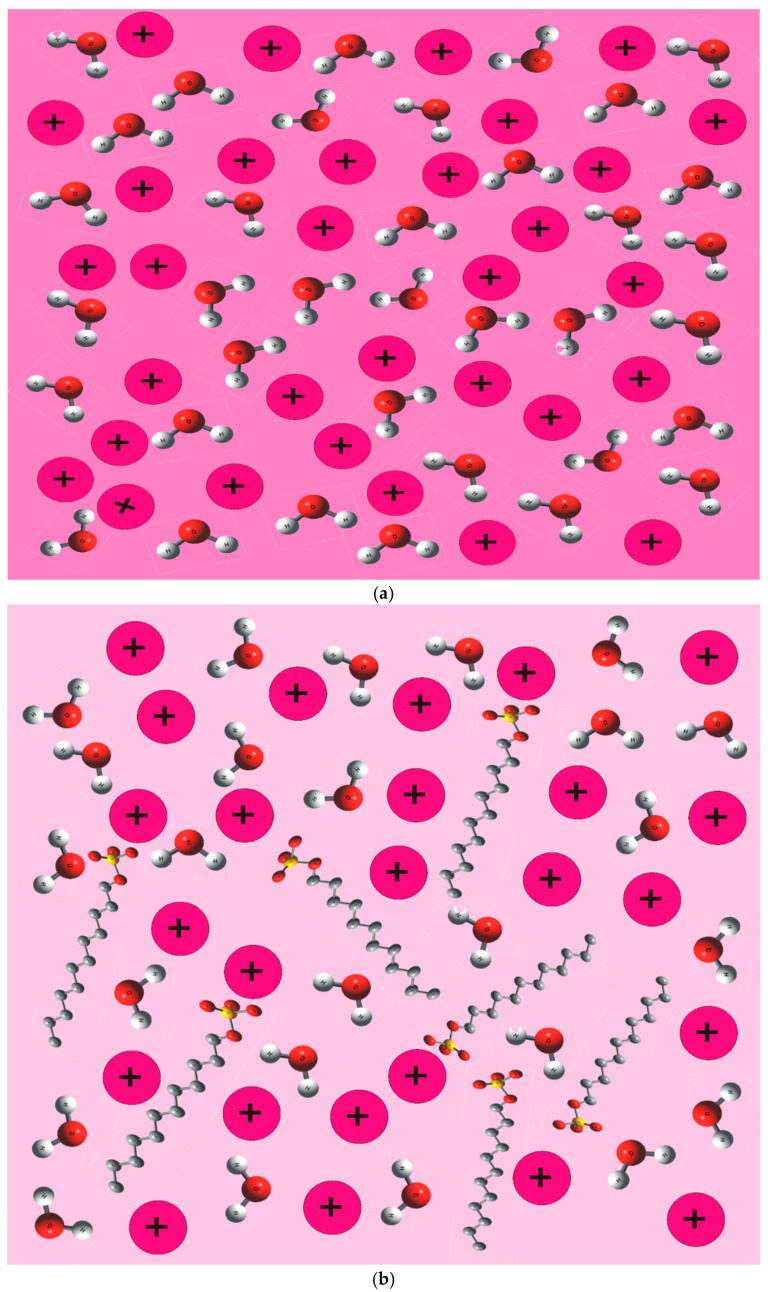

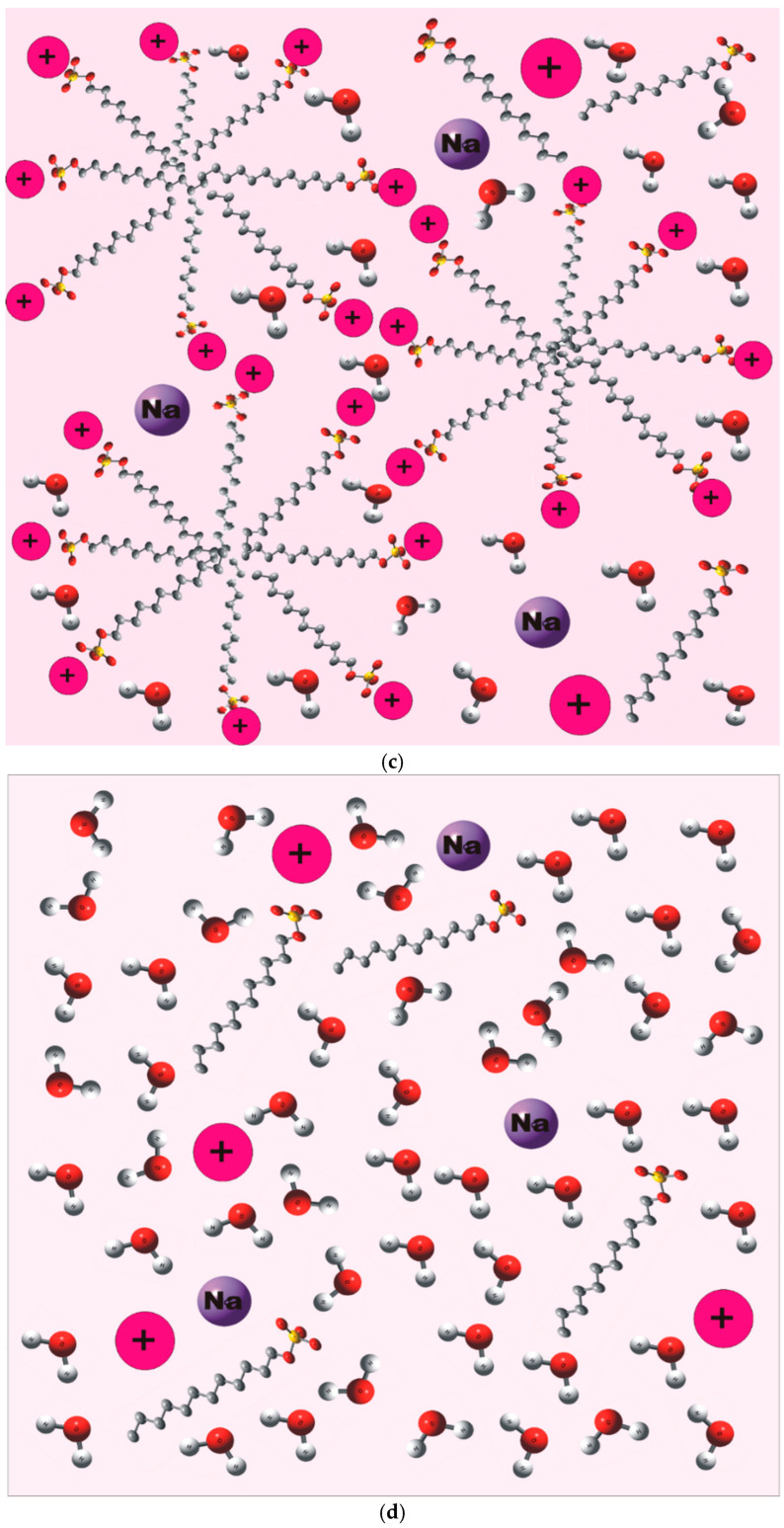
(**a**) Cobalt solution in aqueous phase. (**b**) Interaction of Co^2+^ with surfactant in water. (**c**) Adsorption of metal ions on the micelles surface. (**d**) Permeate solution.

In a micellar system, the micelles have an ability to trap the pollutant molecule; therefore, the true solution was converted to a colloidal solution form and can be easily removed. This study is basically designed as an application of the micellar-enhanced ultrafiltration process in order to remove Co^2+^ from aqueous media. Two anionic surfactants, i.e., SO and SDS, were used along with 10,000 molecular weight cutoff (MWCO) having a thin-film composite [26]. This study represents the optimal conditions to increase the removal efficiency of Co^2+^. Scheme 2 was drawn on Corel draw 12, at a resolution of 300 × 300 dpi, size of 6009 and 5914 pixels, width and height of 20.03 and 19.71 inches. The following represents Co^2+^ removal by the MEUF process: (a) presence of metal ions in aqueous media, (b) describes the surfactant addition in aqueous solution of Co^2+^, (c) displays how Co^2+^ molecules are adsorbed at the surface of micelles and (d) exhibits the filtration process where most of molecules of metal (Co^2+^) were removed and the permeates have too low Co^2+^ concentration.

#### 2.2.1. Rejection Percentage

The study of factors below calculates the best rejection percentage of metal ions by micellar media of SO and SDS.

##### Surfactant Concentration

**Figure 4:**
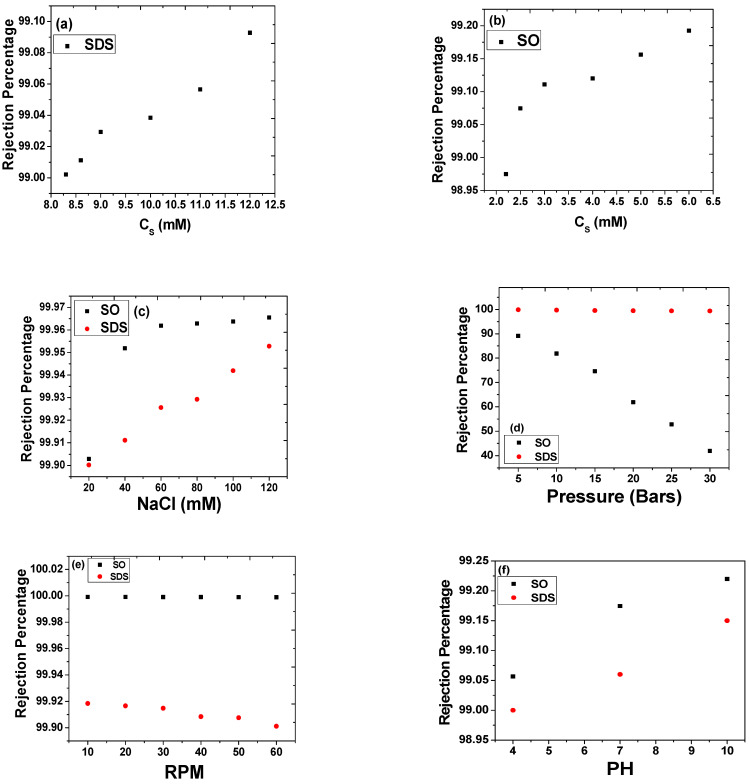
Rejection percentage (R%) of (**a**) SDS concentration (C_SDS_), (**b**) SO concentration (C_SO_), (**c**) electrolyte concentration (NaCl) of SDS and SO, (**d**) pressure, (**e**) RPM, (**f**) pH.

By keeping the CoCl_2_ concentration constant (0.01 mM), transmembrane pressure of 5 bar, 25 °C temperature, speed of 10 RPM and in the absence of any electrolytes, the influence of the variable concentration of both anionic surfactants, in the range of 2–6 and 8–12 mM (for SO and SDS respectively) on the removal efficiency of metal Co^2+^ was studied. The concentration of both surfactants was selected at or above the CMC. An increase in the concentration of the surfactant resulting in enhanced rejection efficiency by SDS and SO is graphically represented in Figure 4a,b. The effect of the concentrations of surfactants on the removal percentage is given in Table 2.

**Scheme 3:**
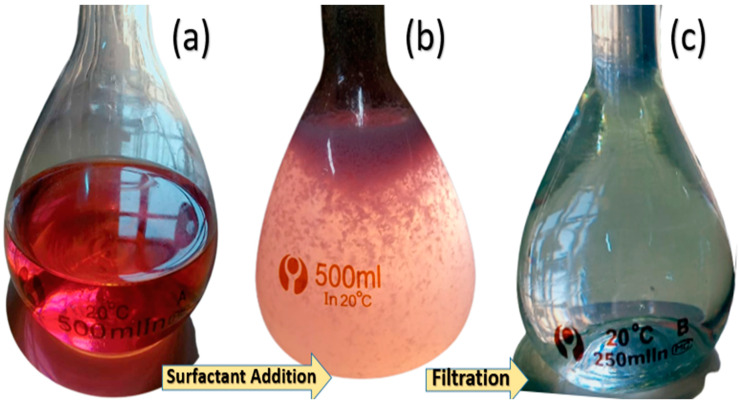
A pictorial insight into the ion–surfactant interaction and micelles formation: (**a**) solution of Co^2+^, (**b**) micelles formation, (**c**) after filtration.

Maximum rejection was observed to be 99.19 for SO and 99.09% in the case that SDS was achieved. The removal of Co^2+^ ions increases by the rising concentration of the surfactant because when the concentration is in the pre-micellar region, the surfactant monomers start forming complexes with the metals by the surface-adsorption phenomenon, [26,45] while in the post-micellar region, a large number of ions become adsorbed at the micellar surface due to the availability of more surfactant monomers to bind cations due to the electrostatic interaction between the micelles and the oppositely charged metal ions. So, a small-sized molecule which can be normally rejected by a membrane can bind to the micelles-charged polar head group and therefore enhance the size of the metal ion by ionic interactions, and obviously a large molecular weight can easily be retained by the membrane [9]. When the micelles form above the CMC level, at this stage, due to a negative charge on the polar head of both the SDS and SO, the surfactant interacts with the oppositely charged, i.e., positively charged, metal ions and therefore the metal ions become attached at the micelles’ surface or polar head groups [46]. A pictorial insight into the ion–surfactant interaction and micelles formation can be seen in Scheme 3a, solution of Co^2+^ (b) micelles formation (c) after filtration.

##### Concentration of Electrolyte

The influence of different concentrations of electrolytes, keeping all other parameters constant ranging from 20 to 120 mM, was studied. By increasing the concentration of electrolytes, an increase in the rejection percentage was observed in Figure 4c in the cases of SDS and SO, respectively. The electrostatic force of interaction takes place in between NaCl, Co^2+^ and the membrane [47]. NaCl as an electrolyte was observed to act as a hindrance for the Co^2+^ passageway and as a result, a significant removal efficiency of Co^2+^ was observed [48]. An increasing concentration of free counter ions results in decreasing the forces of repulsion present among the charged (polar) head groups of micelles, which are fighting against the aggregation of surfactant monomers, and hence a decrease in the critical micellar concentration and an increase in the micelles aggregation number were observed [40,49]. The effect of electrolytes on the rejection percentage is summarized in Table 3, and maximum R% of 99.95% for SDS and 99.97% for SO was observed. The study of the electrolyte effects is necessary, as they are normally present in aqueous systems. In MEUF, in the presence of electrolytes, due to the neutralization of charges, the CMC decreases. The CMC of ionic surfactant decreases, as the concentration of salt increases in the solution. The reason behind this is the compression in the electric double layer, so it may result in a reduction in the electrostatic attraction in ions and micelles [45].

##### Effect of Transmembrane Pressure

A rise in the pressure or increase in the transmembrane pressure, which is basically the pressure gradient, exists across the feed. The permeate side resulted in increasing the rejection coefficient. It is actually the driving force in MEUF, and is much lower, compared to the nano-filtration process for the removal of small-sized molecules. This increasing pressure can overcome the resistance to flow across the membrane, so more solution was observed to pass through the pores of the membrane [50]. Hence, the rejection percentage decreases by increasing the transmembrane pressure [51,52]. At very low pressure, the maximum Co^2+^ removal was examined, and a decline in the rejection was studied by increasing the pressure for both cases of SDS and SO; 99.08% and 89.01% efficiency was found, respectively. A graphical explanation is given in Figure 4d, and data are given in Table 4. For the removal of metal ions, the transmembrane pressure is found to be a major component to control the MEUF efficiency. At high pressure, the resistance to flow across the membrane decreases [45].

##### RPM

Revolutions per minute (RPM) was observed at 10, 20, 30, 40, 50 and 60 RPM values. Micelles with adsorbed metal ions, being larger in size, are capable of being filtered by an ultrafiltration 10,000 MWCO membrane [53]. The de-micellization occurs at high speed, and hence a decline in the Co^2+^ removal was observed. Therefore, the molecules of metal ions can easily be penetrated by membranes as shown in Figure 4e, and the results are tabulated in Table 5. At low RPM, the greater rejection percentage obtained was 99.91% in the case of SDS, whereas it was 99.99% for SO [26].

##### pH

To calculate the effect of pH of Co^2+^, the filtration process was carried out at variable pH. The pH values chosen were 4, 7, and 10, and solutions were prepared in a micellar media of both surfactants by keeping all other parameters constant. An increase in the R% was observed at a high pH value, and the results have close agreement with the literature reported values [40]. The effect of pH also depends on the nature of the surfactants. The reason behind this is that, at a low pH, competition between the H^+^ and the metal cations begins and a decline in the R% is observed. In the case of anionic surfactants, protonation can decrease the cations’ interaction with micelles at a low pH, but at higher pH, large numbers of free groups are available for binding cations. Maximum R% was observed at a pH of 10, so this pH was selected for further work. The R% was obtained as 99.15% and 99.21% for SDS as well as SO, respectively, and can be seen in Figure 4f and in Table 6. Therefore, pH is a very essential factor for metal ions removal [40,54].

#### 2.2.2. Permeate Flux

##### Concentration of Surfactants

**Figure 5:**
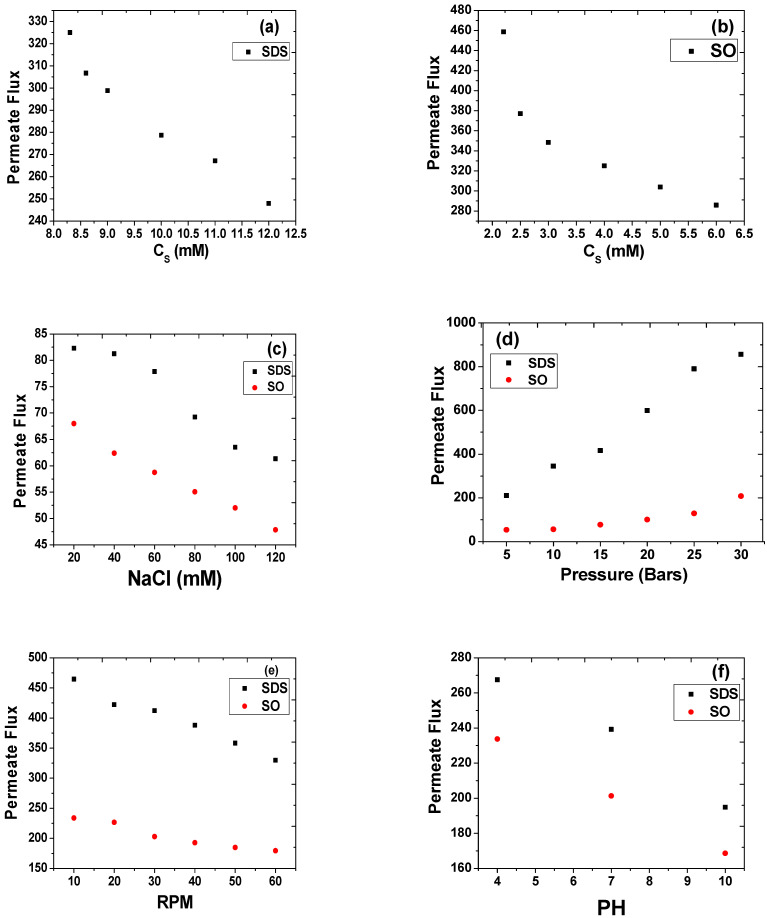
Permeate flux (J) of (**a**) SDS concentration (C_SDS_) (**b**) SO concentration (C_SO_) (**c**) electrolyte concentration (NaCl) of SDS and SO (**d**) pressure (**e**) RPM (**f**) pH.

The permeate flux of Co^2+^ was studied at a constant Co^2+^ concentration (0.01 mM), transmembrane pressure of 5 bar, at 25 °C, at 10 RPM and in the absence of any electrolyte (NaCl) to observe the influence of different concentrations of anionic surfactants, i.e., C_so_ and C_SDS_, in the range of 0 to 6 and 0 to 12 mM, respectively. A variation in the permeate flux with the increasing concentrations of surfactant is shown in Figure 5a,b. The removal efficiency increases with the surfactant concentration because below CMC, the monomers of surfactants form complex with the metals, which can easily pass through the pores of membrane, but a high concentration above CMC or at the CMC point, micelle formations occur, providing more reactive sites by more surface area for the binding of metal ions, and hence more time is required for the penetration through the membrane. So, a reduction in the flux by an increase in the concentration of surfactants was observed. The reason is particularly the gel layer formation at the surface of the membrane, which can cause a resistance to flow across the membrane, and hence a decrease in permeate flux was observed [45,46]. The data are presented in Table 2.

##### Concentration of Electrolyte

A decline in the permeate flux was observed in the presence of electrolytes for both cases of anionic surfactants, which is plotted in Figure 5c and the data are given in Table 3. An explanation to this trend is that in the presence of electrolytes, an increase in the metal ions adsorption takes place, resulting in increased aggregation [45]. The effects of enhanced polarization concentration on the surface of the membrane due to the greater concentration of electrolytes consequently drop the value of the permeate flux. The permeate flux also depends on the type of solute and the concentration as well [22,52,55].

##### Effect of Transmembrane Pressure

At higher pressure, a rise in the permeate flux takes place by focusing the diffusion model of solutions. An increasing direct relation can be seen in Figure 5d and in Table 4. An increasing pressure causes an increase in the driving force, which is capable of overcoming the membrane resistance and osmotic pressure. Hence, this process forces more solution to flow across the membrane, and therefore a high permeate flux was observed. Mass transfer was observed to be low or constant at a very low pressure but increases speedily by increasing pressure [38,56]. At the CMC, a larger number of surfactant monomers arrange themselves to form micelles at the surface of the membrane, and hence provide more available sites to interact with metal cations [45]. High pressure results in the decrease in the resistance across the membrane [57].

##### Effect of RPM

As the loaded micelles have a large size, they therefore can be easily filtered by the 10,000 MWCO ultrafiltration membrane [53], but as the speed increases, the process of de-micellization takes place and reduces the efficiency of this process. At a high RPM value, the solution penetration across the membrane pores rises, which is graphically plotted in Figure 5e as well as in Table 5. Hence, a decline in the permeate flux was observed with RPM [26].

##### Effect of pH

The anionic surfactants protonation was observed at low pH, which decreases the cations’ interactions with micelles. Instead, there are large number of free groups that are accessible for cation binding. In this study, pH 10 was found to be suitable for effective rejection [58]. As the pH value increases from 4–10, the permeate flux value decreases because of the factor of concentration polarization as shown in Figure 5f and in Table 6 [26,40].

## 3. Materials and Methods

### 3.1. Materials

**Scheme 4:**
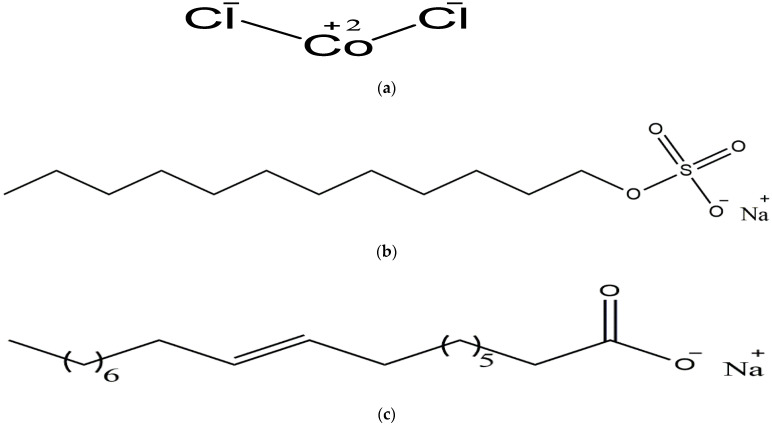
Structures of metal and surfactants, (**a**) cobalt chloride (II), (**b**) sodium dodecyl sulfate (SDS), (**c**) sodium oleate (SO).

Two anionic-nature surfactants, i.e., sodium dodecyl sulfate (SDS) and sodium oleate (SO), were used as a primary surfactant in order to prepare the solution in micellar media and were purchased from Daejung, Korea. Metal salt cobalt chloride (CoCl_2_) (molar mass 129.83 g/mol) was purchased from Sigma Aldrich. The chemicals required along with some details are given in Table 7. A 10,000 MWCO of regenerated cellulose membranes was bought from Amicon Bioseparations EMD Millipore Company, Billerica, MA 01821 in the USA. The process of ultrafiltration was carried out using a stirring cell (Amicon 8400 Millipore, corporation, Burlington, MA, USA). The structures of metal and surfactants are shown in Scheme 4.

### 3.2. Parameter Calculated

#### 3.2.1. Partition Constant and Gibbs Energy of Partition

The molecules of metal cations (Co^2+^) are separated in between the micellar as well as the bulk phase. Therefore, between these two phases, the comparative distribution of Co^2+^ molecules can be quantified as a partition coefficient. The Kawamura equation is given [36,59]:

(1) $$\frac{1}{\Delta A}=\frac{1}{K_{c}\;{\Delta A}_{\infty }\left(C_{M}+C_{s}^{mo}{}_{}\right)}+\frac{1}{{\Delta A}_{\infty }}$$

Here, *C_M_* is the metal ions molar concentration in mold m^−3^, whereas *C_s_^mo^* is the surfactant analytical concentration and can be written/calculated as

(2) $$C_{s}^{mo}=C_{s}-CMC_{o}$$

where *C_s_* indicates the overall molar surfactant concentration, and *CMC_o_* is the *CMC* in the absence of metals. ΔA is the representation for the differential form of absorbance at the experimental level and ΔA_∞_ is constant at the infinite dilution.

The partitioning of metal ions between aqueous and micellar media is governed by the partition law [60]. The K_c_ is the partition constant with units of dm^3^mol^−1^, and K_x_ is the partition coefficient having no unit. K_x_ is the multiple of K_c_ and n_w_, where n_w_ is the number of moles of water per liter. Equation (3) is used for the calculation of Gibbs energy of partition [11,61,62]:

(3) $${\Delta G}_{p}=-RTlnk_{x}$$

#### 3.2.2. Binding Constant and Gibbs Energy of Binding

The metal ion complexation and micelle is a dynamic process represented as given below:

(4) $$D+S_{mic\;}\overset{k_{b}}{\Leftrightarrow }\;DS_{mic}$$

(5) $$k_{b=}\frac{\left[DS_{mic}\right]}{\left[D\right]\left[S_{mic}\right]}$$

The concentrations of Co^2+^, micelles and Co^2+^/surfactant complex are denoted as [*D*], [*S_mic_*] and [*DS_mic_*], respectively. By using the Benesi–Hildebrand equation (Equation (6)), the value of the binding constant (*K_b_*) can be calculated:

(6) $$\frac{dC_{m}}{\Delta A}=\frac{1}{k_{b}\Delta \varepsilon C_{s}{}^{mo}}+\frac{1}{\Delta \varepsilon }$$

“*d*” is the optical path length (10 mm), “*C_m_*” for Co^2+^, concentration “Δ*A*” is the differential absorbance, “Δε” for differential molar absorptivity, “*C_s_^mo^*” is the analytical surfactant concentration and “*K_b_*” is for the binding constant. The change in Gibbs free energy of the binding of the Co^2+^-surfactant is calculated using Equation (7) [63]:

(7) $${\Delta G}_{b}=-RTlnK_{b}$$

#### 3.2.3. Rejection Coefficient (R%)

In the MEUF process, the metal molecules are adsorbed at the surface of micelles, and these micelles, being larger in size, are retained over the membrane surface and, subsequently, rejected by the ultrafiltration membrane. The value of the rejection coefficient is calculated by Equation (8):

(8) $$R\left(\%\right)=\left[1-\frac{C_{P}}{C_{F}}\right]\times 100\;$$

In Equation (8), *C_P_* and *C_F_* show concentrations of pollutants in the permeate as well as in the feed, respectively.

#### 3.2.4. Permeate Flux (J)

A variation in the permeate flux is also a way to find out the efficiency of this process. It was noticed that a decline in the flux decreases with time due to the formation of micellar layers at the membrane [22,26]. The permeate flux is calculated as

(9) $$J=\frac{V}{t\times A}$$

In Equation (9), “*V*” represents the volume of permeate solution, “*t*” is the time taken by the ultra-filtration experiment, and “*A*” is the effective area used of the membrane.

## 4. Experimental Methods

### 4.1. Solution Preparation

A UV/visible double beam spectrophotometer from Peaks C-8200S, United States of America, was utilized, which was provided by a Xenon lamp, in order to record the absorption spectra [64,65].

Two anionic surfactants were selected for this work, as both are cheaper and have better interaction with metal cations than any other surfactant due to opposite changes for surfactants and metal ions. A chain of ternary solutions of surfactants having various concentrations in the range of sub micellar to micellar regions at a constant concentration of Co^2+^ 1 × 10^−5^ M was set in distilled water. By using a solution of pure CoCl_2_, the said solutions were diluted in order to create constant concentration. For dynamic equilibrium, all solutions were left overnight or for 24 h. The distilled water was placed in the reference cell in case of simple absorption spectra, whereas the solution of CoCl_2_ was placed as reference. However, in all the cases, water/Co^2+^/surfactant as a ternary solution was used in the sample cells [65,66,67].

### 4.2. Micellar Enhanced Ultrafiltration (MEUF)

The solutions of two surfactants, anionic in nature, i.e., sodium dodecyl sulfate (SDS) has the sulfate group, and sodium oleate (SO) has the COO^−^ group, were used to remove Co^2+^ by (regenerated cellulose) membranes with 10,000 molecular weight cut to carry out the ultrafiltration process, including the agitated cell setup from Amicon 8400 Millipore, USA. Good quality distilled water was used in order to soak membranes to remove water-soluble species. The washing of membranes was performed with distilled water after each filtration experiment. At room temperature, the operating pressure was varied, ranging from 5 to 30 bars. Feed solutions and permeate were analyzed by a UV/visible double beamspectrophotometer with an optical length of 1 cm. A number of affecting factors, such as surfactant concentration, Co^2+^ concentration, concentration of electrolyte, trans-membrane pressure, pH and RPM, were noted. Due to the electrostatic force of attraction, the metal ions were adsorbed on the surface. Normally, a surfactant whose charge is opposite to the metal ions attains more retention. So, anionic surfactants, such as SDS and SO, were used as suitable for binding and removal purpose [41].

## 5. Conclusions

The removal of metal ions from an aqueous system was carefully studied via the MEUF process in micellar media of anionic surfactants, i.e., sodium dodecyl sulfate (SDS) and sodium oleate (SO). The adsorption of metal ions at the surface was studied. The calculations for partition (K_x_) and binding constants (K_b_) and their respective free energy of partition and binding (ΔG_p_ and ΔG_b_ kJmol^−1^) helped a lot to find out the extent of the binding or interaction of Co^2+^ with the surfactant, and ΔG_p_ and ΔG_b_ were found to be −29.50 and −19.38 kJmol^−1^ for SDS and −23.95 and −12.67 kJmol^−1^ in the case of SO. After that, MEUF was performed, so the percentage removal was quantitatively discussed in terms of the rejection percentage as well as permeate flux, which were calculated under variable experimental conditions. Several factors affecting the efficacy of MEUF process were also observed. So, the effect of a single variable was measured by keeping all other parameters constant to investigate the finest condition for the removal of metal ions. The rejection of Co^2+^ was studied, ranging from 0 to 12 mM concentration of SDS, and 0 to 6 mM concentration of SO. A maximum rejection of 99.09% and 99.19% was attained by SDS and SO, respectively. A maximum rejection of 99.95% and 99.97% was obtained by using 20–100 mM concentration of NaCl, at 5–30 bars of pressure; the maximum rejection was observed to be 99.8% and 89.1% with SDS and SO, respectively. The highest rejection of 99.918% by SDS and 99.999% by SO was studied by taking RPM values ranging from 10 to 50 and at pH values of 4, 7 and 10. A maximum R% was observed to be 99.15 and 99.21 with SDS and SO, respectively. Resultantly, the rejection percentage was noticed to increase with increasing the surfactant concentration, pH and concentration of electrolyte at low RPM value and pressure, but a decrease in the permeate flux was observed at a high concentration of surfactants and electrolytes at a high pH, whereas low RPM and pressure were found for both cases of SDS and SO. Overall, maximum rejection rates (R%) of 99.95% and 99.99%, whereas a high flux J values of 856 L/hm^2^ and 3262 L/hm^2^ were attained with SDS and SO, respectively.

## Figures and Tables

**Table 1:** Partition constant, K_c,_ partition coefficient, K_x_, binding constant (k_b_), Gibbs energy of partition, ∆G_p_ and Gibbs energy of partition, ∆G_p_ for Co^2+^ in SDS and SO systems.

Surfactants	K_c_ × 10^−4^ (dm^3^mol^−1^)	K_b_ × 10^−5^ (dm^3^mol^−1^)	K_x_ × 10^−5^	ΔG_b_ (kJmol^−1^)	ΔG_p_ (kJmol^−1^)
SDS	0.268	2.50	1.49	−19.38	−29.50
SO	0.003	1.67	0.15	−12.67	−23.95

**Table 2:** Calculation of rejection coefficient (R%) and permeate flux (J) of Co^2+^ against different surfactant concentrations (Cs) at fixed Co^2+^ concentration in feed (C_f_), Co^2+^ concentration in permeate solution (C**_p_**), time of filtration (t), and permeate volume (V**_p_**).

Sr. No.	C_S_ (mM)		C_f_ (mM)	C_p_ (μM)		R %		t (h)		V_p_ (mL)	J (L/hm^2^)	
	SDS	SO		SDS	SO	SDS	SO	SDS	SO		SDS	SO
1	0.0	0.0	0.01	0.113	0.318	98.86	96.81	0.20	0.02	30	349	3262
2	8.3	2.2	0.01	0.099	0.102	99.00	98.97	0.22	0.15	30	324	458
3	8.6	2.5	0.01	0.098	0.092	99.01	99.07	0.23	0.19	30	306	377
4	9.0	3.0	0.01	0.097	0.088	99.02	99.11	0.24	0.20	30	298	348
5	10.0	4.0	0.01	0.096	0.087	99.03	99.12	0.25	0.22	30	278	324
6	11.0	5.0	0.01	0.094	0.084	99.05	99.15	0.26	0.23	30	267	303
7	12.0	6.0	0.01	0.090	0.080	99.09	99.19	0.28	0.25	30	247	261

**Table 3:** Calculation of rejection coefficient (R%) and permeate flux (J) of Co^2+^ using different electrolyte concentration (NaCl) in SDS and SO solutions, at fixed Co^2+^ concentration in feed (C_f_), Co^2+^ concentration in permeate solution (C**_p_**), filtration time (t), permeate volume (V**_p_**).

Sr. No.	[NaCl] (mM)	C_f_ (mM)	C_p_ (μM)		R %		t (h)		V_p_ (mL)	J (L/hm^2^)	
			SDS	SO	SDS	SO	SDS	SO		SDS	SO
1	20	0.1	0.099	0.097	99.90	99.902	0.87	1.05	30	82.2	67.9
2	40	0.1	0.088	0.048	99.91	99.951	0.88	1.15	30	81.2	62.4
3	60	0.1	0.074	0.038	99.92	99.961	0.92	1.22	30	77.8	58.7
4	80	0.1	0.070	0.037	99.92	99.962	1.03	1.30	30	69.2	55.0
5	100	0.1	0.058	0.036	99.94	99.963	1.13	1.38	30	63.5	52.0
6	120	0.1	0.047	0.034	99.95	99.970	1.17	1.50	30	61.3	47.8

**Table 4:** Calculation of rejection percentage (R%) and permeate flux (J) of Co^2+^ using different values of pressure, in SDS and SO solutions at fixed Co^2+^ concentration in feed (C_f_), Co^2+^ concentration in permeate solution (C**_p_**), filtration time (t), permeate volume (V**_p_**).

Sr. No.	Pressure (Bar)	C_f_ (mM)	C_p_ (μM)		R %		t (h)		V_p_ (mL)	J (L/hm^2^)	
			SDS	SO	SDS	SO	SDS	SO		SDS	SO
1	5	0.01	0.01	0.010	99.8	89.1	0.34	1.33	30	210	53
2	10	0.01	0.03	0.018	99.6	81.8	0.20	1.27	30	345	56
3	15	0.01	0.04	0.025	99.5	74.5	0.17	0.92	30	416	77
4	20	0.01	0.05	0.038	99.4	61.8	0.11	0.71	30	598	100
5	25	0.01	0.05	0.047	99.4	52.8	0.09	0.55	30	790	129
6	30	0.01	0.06	0.058	99.3	41.9	0.08	0.34	30	856	208

**Table 5:** Calculation of rejection coefficient (R%) and permeate flux (J) of Co^2+^ against different RPM values in solution of SDS and SO, at fixed Co^2+^ concentration in feed (C_f_), Co^2+^ concentration in permeate solution (C**_p_**), filtration time (t), permeate volume (V**_p_**).

Sr. No.	RPM	C_f_ (mM)	C_p_ (μM)		R %		T (h)		V_p_ (mL)	J (L/hm^2^)	
			SDS	SO	SDS	SO	SDS	SO		SDS	SO
1	10	0.01	0.081	0.081	99.918	99.9991	0.154	0.307	30	464	233
2	20	0.01	0.083	0.087	99.916	99.99912	0.17	0.316	30	422	226
3	30	0.01	0.085	0.094	99.914	99.99905	0.174	0.353	30	412	202
4	40	0.01	0.091	0.097	99.908	99.99902	0.185	0.372	30	387	192
5	50	0.01	0.092	0.103	99.907	99.99896	0.200	0.388	30	358	184

**Table 6:** Calculation of rejection coefficient (R%) and permeate flux (J) of Co^2+^ against different pH values, in solution of SDS and SO at fixed Co^2+^ concentration in feed (C_f_), Co^2+^ concentration in permeate solution (C**_p_**), filtration time (t), permeate volume (V**_p_**).

Sr. No.	pH	C_f_ (mM)	C_p_ (μM)		R %		t (h)		V_p_ (mL)	J (L/hm^2^)	
			SDS	SO	SDS	SO	SDS	SO		SDS	SO
1	4	0.01	0.08	0.078	99.00	99.05	0.26	0.30	30	267	233
2	7	0.01	0.09	0.082	99.06	99.17	0.3	0.35	30	239	201
3	10	0.01	0.01	0.094	99.15	99.21	0.36	0.425	30	194	168

**Table 7:** Purity, supplier, CAS registry number, and maximum wavelength of chemicals used.

Chemicals	CAS Reg. No.	Purity	Molecular Mass (gmol^−1^)	Supplier	Formulae	λ_max_	
						This Work	Literature Reported
Cobalt (II) chloride	7646-79-9	97%	129.83	Sigma Aldrich	CoCl_2_	512	512
Sodium dodecyl sulfate (SDS)	151-21-3	99%	288.372	Daejung Korea	NaC_12_H_25_SO_4_		
Sodium oleate (SO)	148-19-1	≥99	304.4	Daejung Korea	C_18_H_33_NaO_2_		

## Data Availability

Not applicable.

## Sample Availability

Samples of the compounds are not available from the authors.
